# Resting-state spontaneous brain activity as a neural marker for suicidal ideation in adolescents with non-suicidal self-injury: a voxel-wise and machine learning study

**DOI:** 10.3389/fpsyt.2025.1671813

**Published:** 2025-11-03

**Authors:** Qianqian Li, Li Qi, Zhishun Gao, Jin Li, Xiaomin Pan, Dongpeng Wu, Jiahua Zhang, Hongping Wang, Yanghua Tian, Kai Wang, Tongjian Bai

**Affiliations:** ^1^ Department of Psychology and Sleep Medicine, The Second Affiliated Hospital of Anhui Medical University, Hefei, China; ^2^ Department of Neurology, The First Affiliated Hospital of Anhui Medical University, Hefei, China; ^3^ The School of Mental Health and Psychological Sciences, Anhui Medical University, Hefei, China; ^4^ Institute of Artificial Intelligence, Hefei Comprehensive National Science Center, Hefei, China; ^5^ Anhui Province Key Laboratory of Cognition and Neuropsychiatric Disorders, Hefei, China; ^6^ Collaborative Innovation Center of Neuropsychiatric Disorders and Mental Health, Hefei, China; ^7^ Anhui Institute of Translational Medicine, Hefei, China

**Keywords:** non-suicidal self-injury, suicidal ideation, adolescents, resting-state fMRI, support vector regression

## Abstract

**Background:**

Non-Suicidal Self-Injury (NSSI) is a primary risk factor for suicide, but objective biomarkers to assess this risk are urgently needed. The “prefrontal-limbic dysregulation” model provides a neurobiological framework for self-injurious behaviors. This study aimed to identify resting-state neural markers of suicidal ideation severity in adolescents with NSSI and to build a predictive model for individualized risk assessment.

**Methods:**

We recruited 64 adolescent psychiatric inpatients with NSSI. Suicidal ideation was measured using the Beck Scale for Suicide Ideation (BSI). Resting-state functional MRI (rs-fMRI) was used to measure spontaneous brain activity via the amplitude of low-frequency fluctuation (ALFF). We performed a whole-brain correlation analysis between ALFF and BSI scores. A support vector regression (SVR) model was then developed using the identified neural feature to predict individual BSI scores.

**Results:**

A significant negative correlation was found between BSI scores and ALFF values in the left Middle Frontal Gyrus (MFG). Lower spontaneous activity in this region was associated with more severe suicidal ideation. The SVR model, based on the left MFG ALFF values, successfully predicted individual BSI scores with significant accuracy (r = 0.492, p < 0.001), a finding confirmed by permutation testing.

**Conclusion:**

Diminished resting-state activity in the left MFG is a key neural correlate of suicidal ideation severity in adolescents with NSSI. The functional activity of the left MFG is a promising biomarker for suicide risk assessment and may serve as a potential target for novel neuromodulatory therapies in this high-risk population.

## 1 Introduction

Non-Suicidal Self-Injury (NSSI) is defined as the intentional, self-inflicted destruction of body tissue without suicidal intent (1). Common manifestations include cutting, burning, hitting, and scratching the skin (2, 3). Distinguished by its unique intent, frequency, and lethality, NSSI is formally recognized in the Diagnostic and Statistical Manual of Mental Disorders, 5th Edition (DSM-5) as a “Condition for Further Study” (1). With a lifetime prevalence of 17–23% in community adolescent samples and as high as 50% in adolescent inpatient populations, NSSI constitutes a significant public health concern (4–8).

The most severe clinical ramification of NSSI is its robust association with suicidality (9). A substantial body of evidence identifies NSSI as one of the most potent and consistent predictors of future suicide attempts, surpassing other established risk factors such as depression and hopelessness (10, 11). Individuals with a history of NSSI face a nearly 30-fold increased risk of attempting suicide compared to the general population, and up to 70% of adolescents who engage in NSSI report at least one lifetime suicide attempt (12, 13). The fluctuating and often concealed nature of suicidal ideation further complicates timely identification and intervention (14, 15). Consequently, there is an urgent need to develop objective, biologically-based markers for precise risk stratification within this high-risk cohort (16, 17).

Resting-State Functional MRI (rs-fMRI) provides a critical neurobiological lens for understanding self-injurious behaviors. The “prefrontal-limbic dysregulation” model is a widely accepted framework positing a shared underlying mechanism for both NSSI and Suicidal Thoughts and Behaviors (STBs) (18–20). This model theorizes that self-injurious behaviors arise from an imbalance between hyper-responsive subcortical limbic regions implicated in the generation of intense negative affect (e.g., amygdala, striatum) and hypo-functional Prefrontal Cortex (PFC) regions responsible for top-down cognitive control and emotion regulation (19, 21, 22). Converging evidence of reduced gray matter volume, attenuated activation, and compromised white matter integrity within this network in both NSSI and STB populations underscores their shared neural correlates (19, 21, 23, 24). Within this circuitry, the PFC serves as the central hub for top-down regulation (25, 26). The lateral PFC, in particular, is integral to emotion regulation strategies and plays a pivotal role in key cognitive control functions such as goal-directed thought, behavioral inhibition, and working memory (22, 27). Therefore, diminished spontaneous activity in this region may signify attenuated top-down control, thereby facilitating the intensification of suicidal ideation.

Although previous research has linked abnormal activity in frontal regions to self-injury and suicidality—observing reduced spontaneous activity in adults and adolescents with a history of suicide attempts (28, 29), demonstrating its utility in classification models (29), and gathering indirect support from meta-analyses and electroconvulsive therapy studies (30–32)—this body of work has notable limitations. Studies have often focused on adult populations or adolescents with a history of suicide attempts, rather than specifically targeting the high-risk NSSI adolescent cohort prior to an attempt. Furthermore, the reliance on classification models, which distinguish between discrete groups, precludes the ability to predict the severity of suicidal ideation along a continuum. These shortcomings limit their clinical applicability for nuanced, prospective risk stratification in adolescents with NSSI.

To address these gaps, the present study recruited a cohort of adolescents with NSSI to identify alterations in resting-state spontaneous brain activity associated with the severity of suicidal ideation and to construct a regression model capable of predicting individual scores. We hypothesized that, given its critical role in the pathophysiology of suicide, diminished spontaneous activity in specific prefrontal cortical regions would be significantly correlated with suicidal ideation. We further hypothesized that this neural signature could serve as a robust feature in a predictive model, enabling a validated estimation of an individual’s suicidal ideation severity.

## 2 Materials and methods

### 2.1 Participants

A total of 64 adolescent psychiatric inpatients were recruited from the Department of Psychology and Sleep Medicine at the Second Affiliated Hospital of Anhui Medical University between February 2023 and November 2024. The inclusion criteria were as follows: (1) a primary diagnosis of a mood or behavioral disorder based on the International Classification of Diseases, 10th Revision (ICD-10), confirmed independently by two board-certified psychiatrists; (2) age 18 years or younger; (3) education level of primary school or higher; (4) right-handedness; and (5) presence of self-injurious behavior meeting the proposed criteria for NSSI in the DSM-5. Exclusion criteria were: (1) comorbid neurological or systemic diseases, a history of head trauma, or other significant medical conditions; (2) pregnancy or lactation; (3) a history of alcohol or other psychoactive substance use disorders; (4) contraindications to MRI, such as the presence of metal implants; and (5) excessive head motion during MRI scanning (defined as maximum translation > 3 mm or maximum rotation > 3°); and (6) incomplete clinical data. The study was conducted in accordance with the Declaration of Helsinki and was approved by the Ethics Committee of Anhui Medical University (No. 83230422). Written informed consent was obtained from all participants and their legal guardians prior to their inclusion in the study.

### 2.2 Clinical assessments

General demographic information, including sex, age, years of education, and duration of illness, was collected for all participants. The severity of depression and anxiety was assessed using the 24-item Hamilton Depression Rating Scale (HAMD-24) (33) and the Hamilton Anxiety Rating Scale (HAMA) (34), respectively. The Adolescent Non-Suicidal Self-Injury Assessment Questionnaire (ANSAQ) was administered to evaluate the functions and underlying motivations of self-injurious behaviors. We further collected information on the total number of self-injury episodes within the preceding two weeks. Suicidal ideation was evaluated using the Chinese version of the Beck Scale for Suicide Ideation (BSI) (35). Originally developed by Beck in 1979 based on clinical experience and theoretical research (36), the BSI is one of the most widely used tools for assessing suicidal ideation in clinical and research settings worldwide (36). The Chinese version was adapted by Li et al. to better suit the local cultural context, and its reliability and validity were subsequently tested (37). The BSI is a 19-item self-report scale that evaluates the severity of suicidal ideation, with total scores ranging from 0 to 38. The scale assesses various aspects of suicidality, including the intensity of active and passive suicidal desires, specific characteristics of the ideation (such as frequency and duration), the extent of preparation for a potential attempt, and deterrents to suicide (38).

### 2.3 MRI data acquisition

All MRI scans were performed on the same 3.0 Tesla Siemens Verio scanner at the Department of Radiology, the Second Affiliated Hospital of Anhui Medical University. Participants were positioned supine in the scanner and instructed to keep their eyes closed, remain awake, and minimize head movements. To attenuate scanner noise, all participants were provided with earplugs. Resting-state fMRI data were acquired using 250 echo-planar imaging (EPI) sequences, yielding a total scan duration of 10 minutes and 9 seconds. The imaging parameters were as follows: repetition time (TR) = 2400 ms, echo time (TE) = 25 ms, flip angle = 90°, matrix size = 64 × 64, field of view (FOV) = 192 × 192 mm², slice thickness = 3 mm, 48 slices in total, and voxel size = 3 × 3 × 3 mm³. High-resolution structural images were obtained using T1-weighted anatomical scans across 176 sagittal slices, with the following parameters: TR = 1900 ms, TE = 2.98 ms, flip angle = 12°, FOV = 256 × 256 mm², slice thickness = 1 mm, and voxel size = 1 × 1 × 1 mm³.

### 2.4 fMRI data preprocessing

Image preprocessing and ALFF calculation were performed using the Resting-State fMRI Data Analysis Toolkit plus (RESTplus, v1.28; http://www.restfmri.net) (39), which is based on Statistical Parametric Mapping (SPM12; www.fil.ion.ucl.ac.uk/spm) (40) software. The preprocessing pipeline included the following steps (1): discarding the first 10 time points to allow for scanner stabilization; (2) slice timing correction and head motion correction; (3) spatial normalization of functional and structural images to the Montreal Neurological Institute (MNI) standard space using the DARTEL algorithm; (4) spatial smoothing with a 6 × 6 × 6 mm Gaussian kernel; (5) removal of linear trends; (6) regression of nuisance covariates, including white matter signals, cerebrospinal fluid signals, global mean signals, and head motion parameters.

### 2.5 ALFF calculation

Following preprocessing, the whole-brain ALFF map was calculated for each participant. For each voxel, the time series was first filtered (0.01–0.08 Hz) and then transformed into the frequency domain using a Fast Fourier Transform to obtain the power spectrum. The square root of the power spectrum was calculated at each frequency, and the average square root across the 0.01–0.08 Hz band was taken as the ALFF value. Finally, each individual’s ALFF map was standardized by dividing it by the mean ALFF value within the whole-brain gray matter mask.

### 2.6 Statistical analysis

Descriptive statistics for demographic and clinical data were calculated using SPSS (Version 25.0). A multiple regression analysis was performed using the General Linear Model (GLM) in SPM12 to investigate the relationship between ALFF values and BSI scores within a whole-brain gray matter mask (excluding the cerebellum). The statistical maps were corrected for multiple comparisons using a False Discovery Rate (FDR) correction at the voxel level with a threshold of p < 0.05, which was based on an initial voxel-level cluster-forming threshold of p < 0.001 and a minimum cluster size of 20 voxels. The xjView toolbox (https://www.alivelearn.net/xjview) (41) was used to identify and report the anatomical locations and peak coordinates of significant clusters. For *post-hoc* analysis, the mean ALFF values were extracted from the significant cluster. The normality of the data distribution was assessed using a Lilliefors test. Based on the test results, a Spearman correlation was conducted to further examine the relationship between the extracted ALFF values and BSI scores. The resulting correlation coefficient (ρ) also serves as an indicator of the effect size. A p-value < 0.05 was considered statistically significant.

### 2.7 Machine learning prediction

A machine learning model was developed in MATLAB 2021b (MathWorks Inc.) using the LIBSVM toolbox (42) to estimate BSI scores from the extracted neural signatures. The mean ALFF values from the significant cluster identified in the whole-brain analysis (i.e., the left MFG) were extracted to serve as the single feature for the prediction model. A support vector regression (SVR) model with a linear kernel was employed to build the predictive model. A Leave-One-Out Cross-Validation (LOOCV) scheme was implemented. In each fold of the LOOCV, one participant was held out as the test set, while the remaining participants constituted the training set. Within each fold, a grid-search approach combined with a 5-fold inner cross-validation was used on the training set to optimize the SVR hyperparameters C (BoxConstraint) and ϵ (Epsilon). The model’s predictive performance was evaluated by calculating the Pearson correlation coefficient (r) and the Root Mean Squared Error (RMSE) between the LOOCV-predicted BSI scores and the actual scores. To test the robustness of our findings, we also explored alternative models and cross-validation schemes (Detailed in **Supplementary Methods**).

### 2.8 Permutation test

To assess the statistical significance of the SVR model’s predictive accuracy, a non-parametric permutation test (5,000 iterations) was performed. In each iteration, the BSI scores (labels) were randomly shuffled among the participants, and the entire LOOCV and prediction process was repeated. This procedure generated a null distribution of correlation coefficients. The p-value was then calculated as the proportion of permutations in which the correlation coefficient was greater than or equal to the one obtained from the real, unshuffled data.

## 3 Results

### 3.1 Demographic and clinical characteristics

The study included 64 adolescents with NSSI. The demographic and clinical characteristics of the participants are summarized in **Table 1**. The mean age of the participants was 14.42 ± 1.65 years, and the sample was predominantly female (79.7%). The average duration of illness was 24.23 ± 20.53 months. Participants reported moderate levels of depression, anxiety, and suicidal ideation, with mean scores for the HAMD-24, HAMA, and BSI being 23.17 ± 11.18, 19.00 ± 8.79, and 20.73 ± 8.32, respectively. Participants engaged in an average of 4.92 ± 2.90 self-injurious behaviors in the two weeks prior to assessment. The majority of participants were receiving psychotropic medication, with SSRIs being the most common treatment (n = 58).

**Table 1:** Demographic and clinical characteristics of the participants (N = 64).

Characteristic	Value
Demographics	
Sex (male/female), n (%)	13/51 (20.3/79.7)
Age (years)	14.42 (1.65)
Education (years)	8.45 (1.47)
Clinical characteristics	
Duration of illness (months)	24.23 (20.53)
HAMD-24 score	23.17 (11.18)
HAMA score	19.00 (8.79)
BSI score	20.73 (8.32)
ANSAQ	
Selfish social	25.34 (6.92)
Negative reinforcement	19.06 (3.09)
Emotional expression	13.84 (3.59)
NSSI frequency	4.92 (2.90)
Medication status, n	
SSRI	58
SNRI	4
NaSSA	2
Antipsychotics	21
Benzodiazepines	6
Non-Benzodiazepines	4
Mood stabilizers	2

Values are presented as mean (SD) for continuous variables and n (%) for categorical variables. NSSI frequency represents the number of self-injurious behaviors in the past two weeks. ANSAQ, Adolescent Non-suicidal Self-injury Assessment Questionnaire. BSI, Beck Scale for Suicide Ideation; HAMD-24, 24-item Hamilton Depression Rating Scale; HAMA, Hamilton Anxiety Rating Scale; NaSSA, Noradrenergic and Specific Serotonergic Antidepressant; SD, Standard Deviation; SNRI, Serotonin-Norepinephrine Reuptake Inhibitor; SSRI, Selective Serotonin Reuptake Inhibitor.

### 3.2 Voxel-wise correlation between ALFF and BSI scores

**Figure 1:**
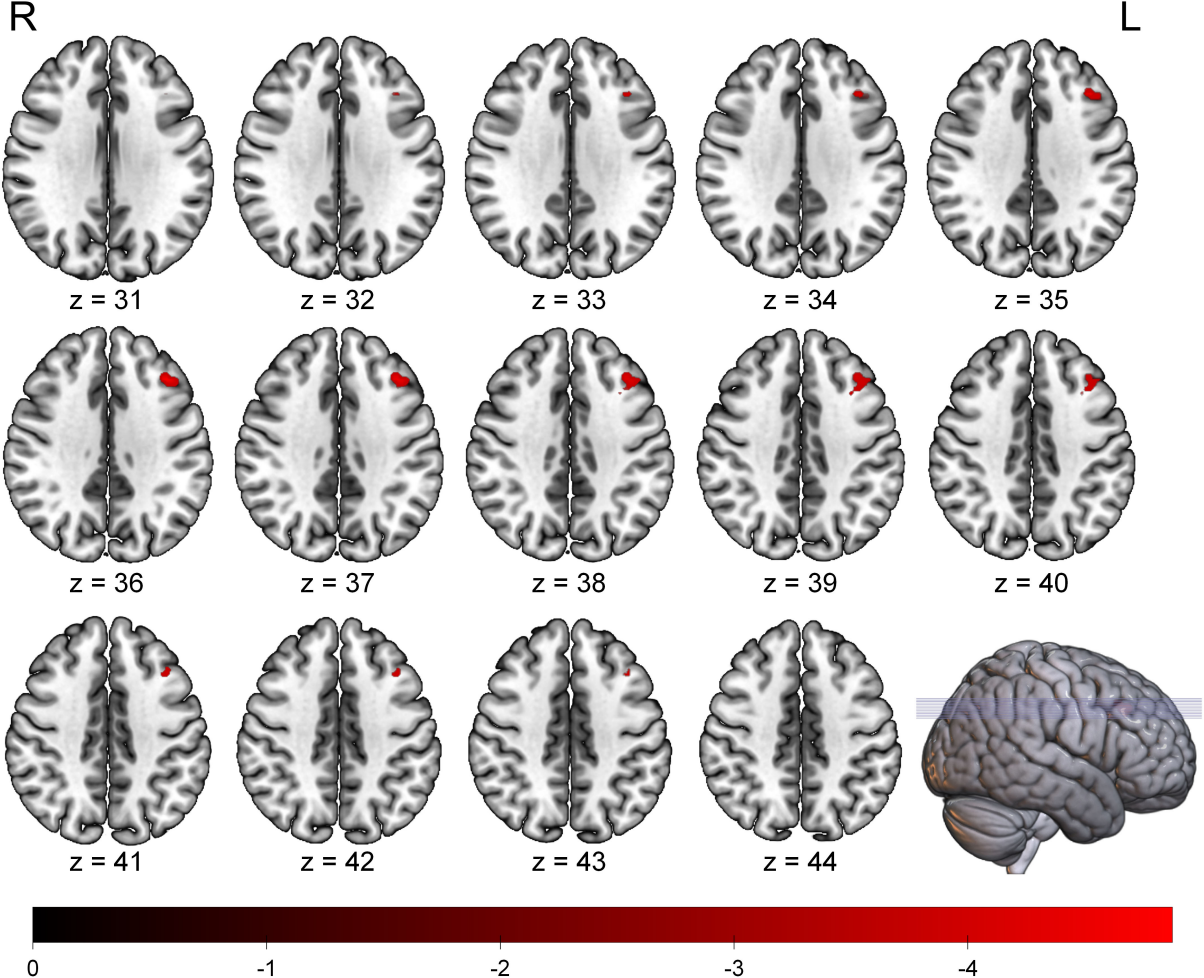
Brain region showing a significant negative correlation between ALFF values and BSI scores in adolescents with NSSI. The significant cluster in the left middle frontal gyrus is overlaid on a standard MNI template brain. The color bar indicates the T-values of the correlation. Statistical maps were set at a threshold of FDR correction (p < 0.05, cluster size > 20 voxels). ALFF, Amplitude of Low-Frequency Fluctuation; BSI, Beck Scale for Suicide Ideation; FDR, False Discovery Rate; L, Left; R, Right.

**Figure 2:**
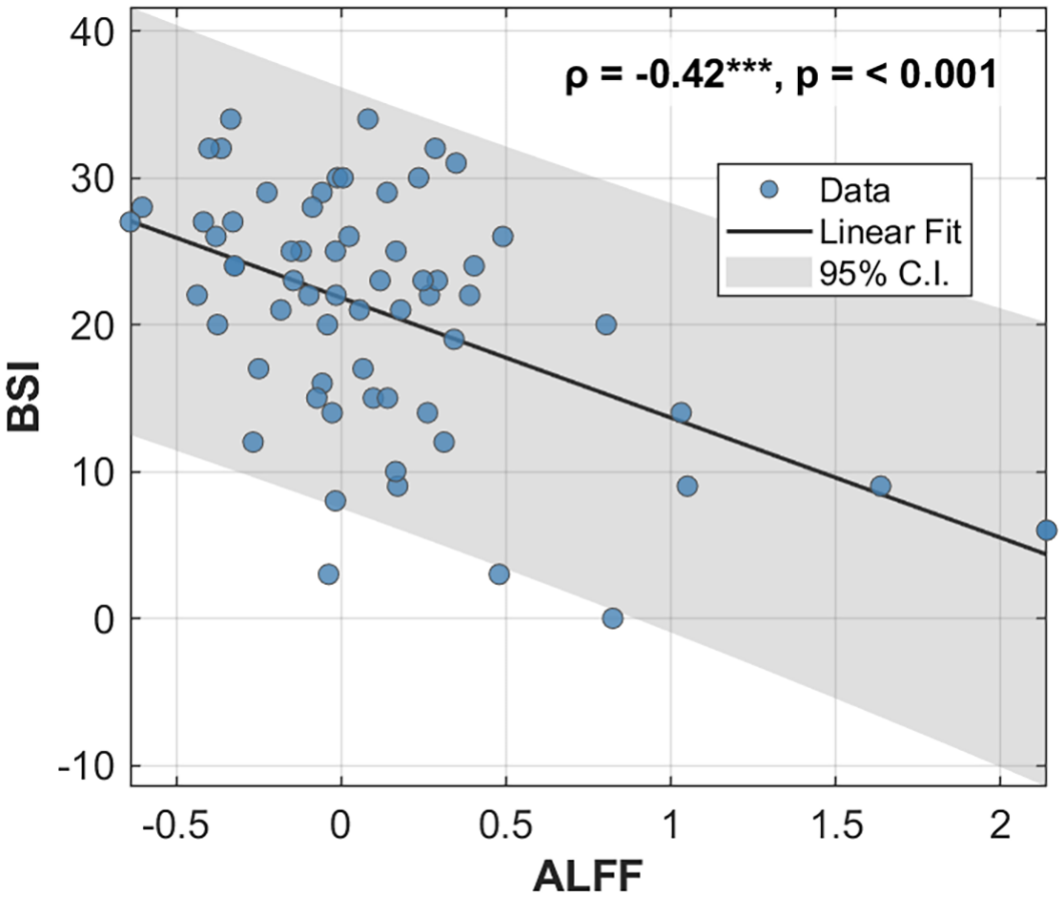
Correlation between ALFF values in the left MFG and BSI scores. Scatter plot illustrating the significant negative correlation between the ALFF values extracted from the left MFG and the BSI scores across all participants. The solid black line represents the linear regression fit, and the shaded gray area indicates the 95% confidence interval. The Spearman correlation coefficient (ρ) and p-value are displayed in the upper right corner. ALFF, Amplitude of Low-Frequency Fluctuation; MFG, middle frontal gyrus; BSI, Beck Scale for Suicide Ideation.

The whole-brain voxel-wise analysis revealed a significant negative correlation between ALFF values and BSI scores in a single brain cluster. This cluster was located in the left Middle Frontal Gyrus (MFG). The detailed statistical information for this cluster is presented in **Table 2**. The anatomical location of this cluster is visualized in **Figure 1**. To further investigate this relationship, the mean ALFF values were extracted from the left MFG cluster for *post-hoc* analysis. A Spearman correlation analysis confirmed a significant negative relationship between the ALFF values of the left MFG and the BSI scores (ρ = -0.42, p < 0.001), as illustrated in **Figure 2**. This indicates that lower spontaneous brain activity in the left MFG is associated with higher levels of suicidal ideation in adolescents with NSSI.

**Table 2:** Brain region showing a significant correlation between ALFF and BSI scores in adolescents with NSSI.

Brain region	Abbreviation	Peak MNI coordinate			Number of voxels	Peak T value
		x	y	z		
Left Middle Frontal Gyrus	L-MFG	-39	27	36	25	-4.940

Results were corrected for multiple comparisons using FDR correction (p < 0.05, cluster size > 20 voxels). ALFF, Amplitude of Low-Frequency Fluctuation; BSI, Beck Scale for Suicide Ideation; MNI, Montreal Neurological Institute; FDR, False Discovery Rate.

### 3.3 SVR prediction performance

**Figure 3:**
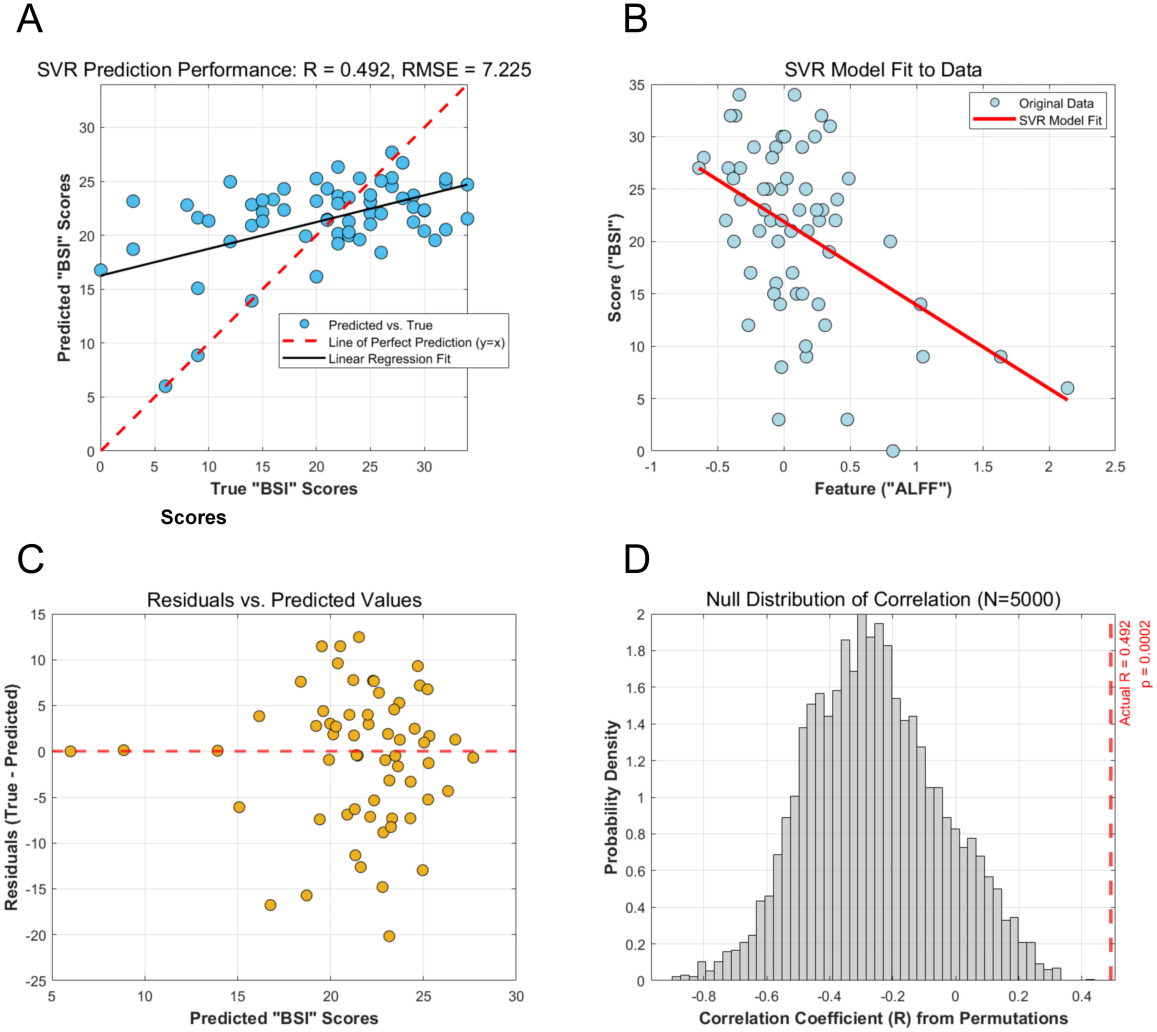
Performance and validation of the SVR model for predicting BSI scores from ALFF values. **(A)** Scatter plot of the SVR model’s predicted BSI scores versus the actual BSI scores. The solid black line represents the linear regression fit, while the dashed red line indicates a perfect prediction (y=x). The Pearson correlation coefficient (R) and RMSE are shown. **(B)** Visualization of the SVR model fit, illustrating the negative linear relationship learned between the ALFF feature and the BSI scores. **(C)** Residual plot showing the difference between actual and predicted BSI scores plotted against the predicted scores. The random distribution of residuals around the zero line indicates no systematic model bias. **(D)** Null distribution of the correlation coefficient from 5,000 permutation tests. The red dashed line indicates the actual correlation coefficient (R = 0.492), which falls far outside the null distribution, demonstrating the statistical significance of the prediction. SVR, Support Vector Regression; BSI, Beck Scale for Suicide Ideation; RMSE, Root Mean Squared Error.

Using the mean ALFF values from the left MFG as the sole feature, the SVR model successfully predicted individual BSI scores. The cross-validated prediction achieved a significant correlation with the actual scores (r = 0.492, p < 0.001), with a RMSE of 7.225 (**Figure 3A**). The model fit demonstrated a clear negative linear relationship between the ALFF feature and BSI scores (**Figure 3B**). Furthermore, the residual plot showed a random distribution of errors around the zero line, indicating no systematic bias in the model’s predictions (**Figure 3C**). The model showed stable performance under 5-fold cross-validation (**Supplementary Figure 1**), with comparable predictive accuracy achieved by alternative linear models, including Linear, Ridge, and Lasso regression (**Supplementary Figure 2**).

### 3.4 Permutation test

The statistical significance of the SVR model’s performance was confirmed by a permutation test with 5,000 iterations. The test revealed that the correlation coefficient obtained from the actual data (r = 0.492) was significantly higher than those generated from the null distribution of randomly shuffled labels (p <0.001). This result demonstrates that the predictive relationship identified by the model is unlikely to be due to chance (**Figure 3D**).

## 4 Discussion

This study aimed to investigate the neurobiological underpinnings of suicidal ideation in adolescents with NSSI and to test the potential of an rs-fMRI-based model for individualized risk prediction. By integrating a voxel-wise whole-brain analysis with a machine learning approach, we identified two core findings. First, within our cohort of adolescents with NSSI, the intensity of resting-state spontaneous brain activity (measured by ALFF) in the left MFG was significantly and negatively correlated with the severity of suicidal ideation (measured by BSI). Second, an SVR model, built upon this neural feature, successfully predicted individual BSI scores with statistically significant accuracy. These findings provide novel neurocircuitry-level evidence for understanding the critical clinical link from NSSI to suicidality and offer a preliminary yet robust foundation for developing objective, interpretable biomarkers for precise risk stratification.

Elucidating the complex clinical relationship between NSSI and suicidal ideation is crucial. Epidemiological and prospective studies have found that up to 70% of adolescents with a history of NSSI report at least one suicide attempt (43). NSSI may be one of the most powerful single predictors of future suicide attempts, with a predictive utility that can exceed traditionally recognized risk factors such as depression and hopelessness (12, 44, 45). The Acquired Capability for Suicide model offers an influential framework for understanding why NSSI may lead to more severe suicidal behaviors (46). According to this theory, an individual must not only desire to end their life but also overcome the fear and pain associated with self-injury, thereby increasing their capacity to engage in suicidal acts (16). NSSI is often used as a strategy to cope with unbearable negative emotions, and this underlying emotional dysregulation is a key factor that may drive suicidal ideation (47). Furthermore, repeated acts of self-injury may reduce an individual’s sensitivity to the pain they cause (48). Over time, this process may normalize self-harm, potentially making it easier to perform more severe or lethal acts (16). Indeed, research suggests a correlation between a higher frequency and longer duration of NSSI and more lethal and frequent suicide attempts (16).

Our central finding is that greater suicidal ideation severity in adolescents with NSSI is associated with diminished resting-state activity in the left MFG. As a metric of spontaneous fluctuations in the BOLD signal, ALFF values directly reflect the intensity of baseline neuronal activity or energy consumption in a given brain region during a task-free state (49, 50). The left MFG is a key component of the dorsolateral prefrontal cortex (DLPFC) (51), which is widely regarded as the “command center” of the cognitive control network (52). This network is responsible for a range of “cold” executive functions, including working memory, planning and execution of goal-directed behavior, attentional allocation, and, critically, the top-down inhibition of maladaptive thoughts and impulses (53–56). The “prefrontal-limbic dysregulation” hypothesis is a cornerstone of neurocircuitry models of psychopathology, positing that symptoms of depression, anxiety, and suicidal behavior arise from impaired regulation of limbic emotion centers, such as the amygdala, by the PFC (18–22). When confronted with negative affect or stress, the prefrontal cognitive control system applies “brakes” to limbic hyperactivity through strategies like cognitive reappraisal, thereby maintaining emotional homeostasis (57, 58). The reduced resting-state activity in the left MFG observed in our study can be interpreted as a marker of compromised functional integrity within this top-down regulatory system. At a behavioral level, this functional deficit may manifest as an inability to effectively suppress intrusive suicidal thoughts (59–61), disengage from negative rumination (62, 63), or successfully reappraise negative cognitions such as hopelessness (64, 65)—all of which are core psychological processes in a suicidal crisis.

This finding converges with a body of prior research, despite some heterogeneity in findings. Previous studies have reported reduced resting-state ALFF associated with suicidal behavior or ideation in other DLPFC subregions, including the medial superior frontal gyrus and precuneus (28, 29). Structural imaging studies have also documented reduced gray matter volume in the DLPFC (66) and weakened functional connectivity (67, 68) in individuals with suicidal ideation. Furthermore, task-based fMRI and fNIRS studies have reported enhanced left DLPFC activation and reduced right DLPFC activation during emotion regulation tasks (65, 69). This may reflect a hemispheric lateralization of DLPFC function in emotion regulation, with the left hemisphere being more involved in cognitive control and approach motivation, while the right is more associated with negative affect and avoidance motivation (70, 71). Collectively, our study contributes specific evidence that diminished intrinsic function of the left MFG may be a core feature of the neurobiological substrate of suicidal ideation within the high-risk population of adolescents with NSSI.

A central goal in psychiatry is the transition from research paradigms based on group-level mean differences to a precision medicine model enabling individualized prediction (72). By employing an SVR model, we successfully predicted the severity of an individual’s suicidal ideation using ALFF values from the left MFG. The model demonstrated not only a significant predictive accuracy (r=0.492) but also a high degree of reliability and interpretability. The use of a LOOCV scheme minimizes model bias, and the permutation test confirmed the robustness of the predictive relationship. This work demonstrates the promise of using functional activity from a specific, theoretically-grounded brain region as a potential biomarker (73).

Several limitations of this study warrant consideration. First, the cross-sectional design precludes causal inferences; it is not possible to determine whether reduced MFG function is a pre-existing trait marker of risk for suicidal ideation or a state-dependent correlate of an acute suicidal episode. Longitudinal studies tracking high-risk adolescents over time are needed to disentangle this relationship (74). Second, our sample size is relatively small and has limited representativeness. This may affect the robustness of the findings and increase the risk of overfitting in the machine learning model. The predominantly female sample limits generalizability to male adolescents, and the recruitment of only inpatients may not represent community-dwelling adolescents with NSSI. Third, the whole-brain correlation analysis did not control for potential confounding variables such as age, sex, illness duration, and medication use. For instance, the female predominance and widespread use of antidepressants in our sample may have altered prefrontal activity, potentially confounding the observed association between ALFF and suicidal ideation. Fourth, the vast majority of our participants were receiving psychotropic medication, which represents a potential confounding factor. Future research should aim to replicate these findings in a medication-naïve sample and incorporate relevant covariates into the statistical model. Fifth, this study lacks a clinical control group, which limits our ability to determine the diagnostic specificity of our findings. The observed alteration in the left MFG’s spontaneous activity might not be specific to suicidal ideation within the NSSI context but could represent a transdiagnostic marker shared with other psychiatric conditions highly comorbid with NSSI, such as major depressive disorder. This potentially overstates the immediate clinical translatability of this finding as a specific biomarker for the NSSI population. Future research should include comparative clinical groups (e.g., major depression patients without NSSI) to clarify the specificity of this neural signature. Finally, this study relied on a single neuroimaging metric, which consequently limited our machine learning model to a single predictive feature. Future investigations should incorporate multimodal imaging approaches to provide a more comprehensive characterization of brain pathology, which may in turn yield higher predictive accuracy (75, 76).

## 5 Conclusion

In conclusion, this study demonstrates that among adolescents with NSSI, a high-risk population for suicide, the resting-state neural activity level of the left MFG is significantly and negatively correlated with the severity of suicidal ideation. We further successfully validated that a machine learning model based on this neural feature can predict individualized suicide risk. Therefore, the resting-state activity of the left MFG not only emerges as a promising potential biomarker but also provides a putative target for the development of more precise neuromodulatory therapeutic strategies.

## Data Availability

The raw data supporting the conclusions of this article will be made available by the authors, without undue reservation.
