# Supplementary material for: Resting-state spontaneous brain activity as a neural marker for suicidal ideation in adolescents with non-suicidal self-injury: a voxel-wise and machine learning study
Source: Front Psychiatry. 2025 Nov 3;16:1671813. doi: 10.3389/fpsyt.2025.1671813 (PMC12620615; doi:10.3389/fpsyt.2025.1671813)
Supplement: Supplementary file 1 [file DataSheet1.pdf]

SUPPLEMENTARY INFORMATION

Resting-State Spontaneous Brain Activity as a Neural Marker for Suicidal Ideation in Adolescents with Non-Suicidal Self-Injury: A Voxel-wise and Machine Learning Study

Li *et al.*

Supplementary Methods

To explore the predictive utility of the left MFG's ALFF value, we implemented and compared four distinct regression models within a Leave-One-Out Cross-Validation (LOOCV) framework. For all models involving hyperparameter tuning, an inner 5-fold cross-validation was performed on each training set to select the optimal parameters that minimized mean squared error (MSE). The models were: (1) Support Vector Regression (SVR), implemented using the LIBSVM toolbox with a linear kernel, where the cost (C) and epsilon (ε) parameters were tuned via a grid-search. (2) Linear Regression, a standard linear model implemented with MATLAB's fitlm function, which has no hyperparameters. (3) Ridge Regression, an L2-regularized linear model implemented with the ridge function, where the regularization parameter (Lambda) was tuned across a logarithmic scale. (4) Random Forest Regression, an ensemble model implemented with the TreeBagger function, where the number of trees was fixed at 100 and the minimum leaf size was tuned.

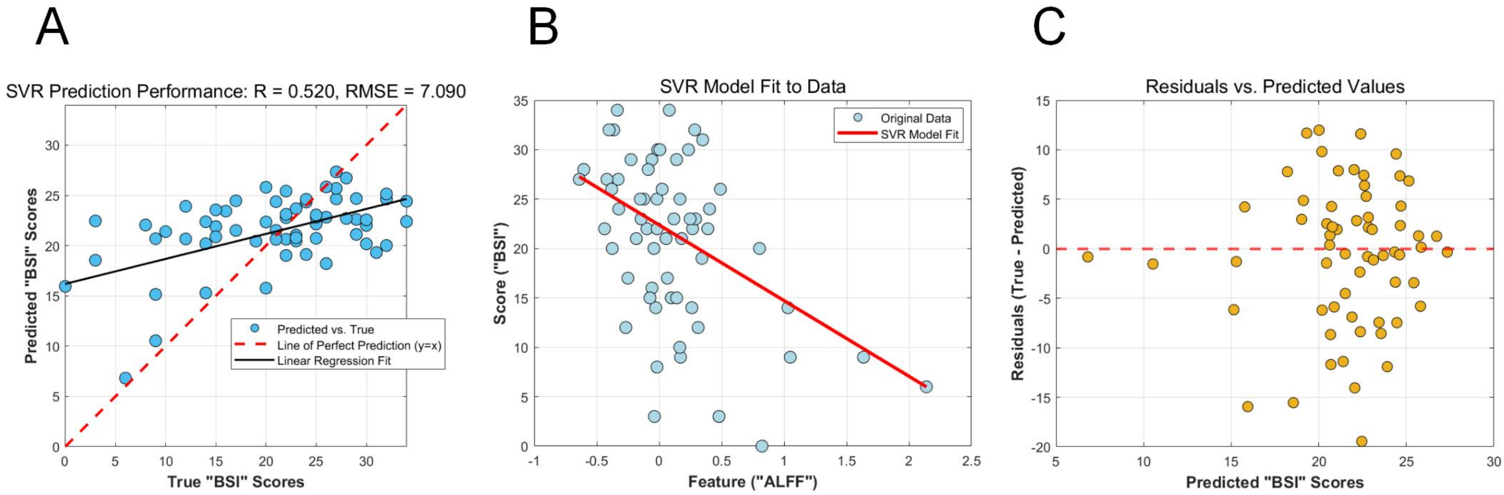

Supplementary Figure 1. Performance and validation of the Support Vector Regression (SVR) model using 5-fold cross-validation. (A) Scatter plot of the SVR model's predicted BSI scores versus the actual BSI scores, demonstrating the model's predictive accuracy (Pearson's R = 0.520, RMSE = 7.090). The solid black line represents the linear regression fit between predicted and true values, while the dashed red line indicates a perfect prediction (y=x). (B) Visualization of the SVR model fit, illustrating the relationship learned by the model between the ALFF feature and the BSI scores. (C) Residual plot showing the difference between actual and predicted BSI scores plotted against the predicted scores. The random distribution of residuals around the zero line indicates no systematic model bias. *Note.* SVR, Support Vector Regression; BSI, Beck Scale for Suicide Ideation; RMSE, Root Mean Squared Error; ALFF, Amplitude of Low-Frequency Fluctuation.

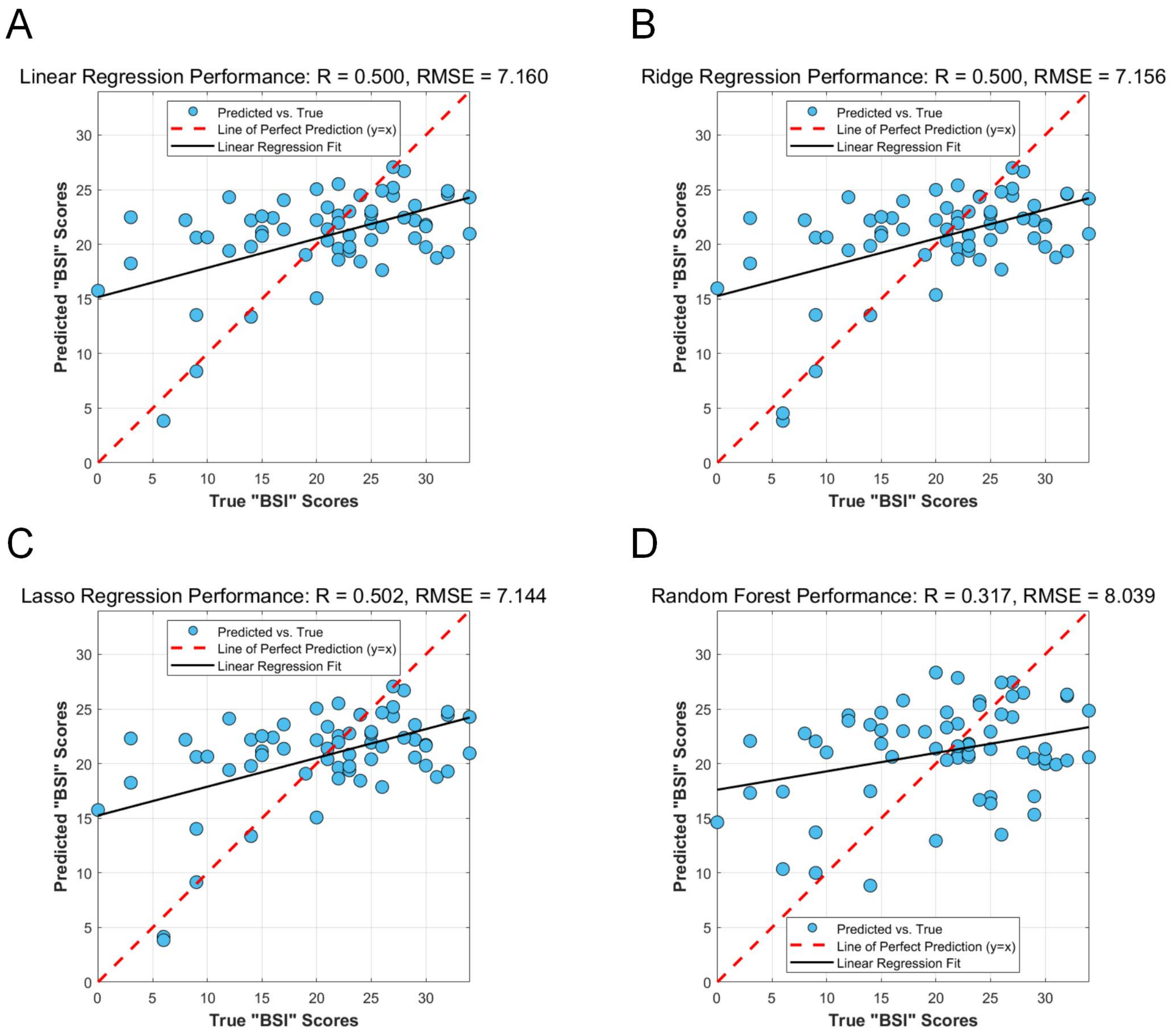

Supplementary Figure 2. Comparison of prediction performance for four regression models using Leave-One-Out Cross-Validation. The figure displays scatter plots of predicted versus true BSI scores for four different machine learning models, allowing for a direct comparison of their performance. For each model, the Pearson correlation coefficient (R) and Root Mean Squared Error (RMSE) are reported. The models are: (A) Linear Regression (R = 0.500, RMSE = 7.160), (B) Ridge Regression (R = 0.500, RMSE = 7.156), (C) Lasso Regression (R = 0.502, RMSE = 7.144), and (D) Random Forest Regression (R = 0.317, RMSE = 8.039).
